# Differential intestinal injury and unchanged systemic inflammatory responses to leg and whole‐body passive hyperthermia in healthy humans

**DOI:** 10.1113/EP092389

**Published:** 2025-02-12

**Authors:** Oliver R. Gibson, Orlando Laitano, Kazuhito Watanabe, José González‐Alonso

**Affiliations:** ^1^ Division of Sport, Health and Exercise Sciences, Department of Life Sciences Brunel University of London Uxbridge UK; ^2^ Centre for Physical Activity in Health and Disease (CPAHD) Brunel University of London Uxbridge UK; ^3^ College of Health and Human Performance, Department of Applied Physiology and Kinesiology University of Florida Gainesville Florida USA; ^4^ Faculty of Education and Human Studies, Department of School Education Akita University Akita Japan

**Keywords:** gut permeability, heat illness, heat therapy, hyperthermia, inflammation, intestinal fatty acid binding protein

## Abstract

Hyperthermia can cause intestinal injury, facilitating endotoxin translocation and an inflammatory response that has been associated with heat illness. However, the potential occurrence of these responses has been incompletely reported during passive hyperthermia, and the independent effect of hyperthermia is equivocal. Furthermore, passive hyperthermia is a feature of heat therapy interventions, with mechanistic understanding developing. This experiment quantified the changes in intestinal fatty acid binding protein (iFABP), a marker of intestinal injury, and cytokine, chemokine and growth factor responses during three different prolonged passive hyperthermia protocols. Eight healthy males visited the laboratory on four counterbalanced occasions to undertake 2.5 h of rest (CON), one‐leg heating (OLH), two‐leg heating (TLH) and whole‐body heating (WBH) via a garment circulating water at 50°C. Plasma concentrations of iFABP and 38 cytokines, chemokines and growth factors were quantified periodically, and core temperature (*T*_core_) was measured continuously. The *T*_core_ increased from baseline in OLH, TLH and WBH (+0.4°C ± 0.2°C, +0.7°C ± 0.2°C and +2.3°C ± 0.4°C, respectively; *P *< 0.05) but remained unchanged in CON. iFABP increased from baseline in WBH only (∆587 ± 651 pg ml^−1^) and was different from CON and OLH in WBH after 2 h (*P *< 0.05). Increased iFABP (∆1085 ± 572 pg ml^−1^) was observed in 50% of participants at the end of WBH, with the other 50% demonstrating no change (∆89 ± 19 pg ml^−1^). All chemokines, cytokines and growth factors were unchanged in all protocols. These data indicate that passive whole‐body hyperthermia, but not lower‐limb hyperthermia, can cause intestinal injury in some individuals without a systemic inflammatory response.

## Abstract (highlights)

- **What is the central question of this study?**
  To what extent does prolonged lower‐limb or whole‐body passive hyperthermia cause intestinal injury and systemic inflammation?
- **What is the main finding and its importance?**
  Intestinal fatty acid binding protein, a marker of intestinal injury, increased only during whole‐body hyperthermia, with augmented concentrations observed in 50% of participants after 2 h. This outcome indicates a potential responder/non‐responder paradigm. Despite this evidence of intestinal injury, concentrations of circulatory cytokines, chemokines and growth factors were unaltered in all passive hyperthermia conditions. These findings expand our knowledge of the gastrointestinal and systemic inflammatory responses to passive hyperthermia and provide insight into the safety of lower‐limb and whole‐body thermal therapy interventions.

## 1 INTRODUCTION

Hyperthermia, whether by passive or exertional stimuli, can cause intestinal epithelial injury, increasing tissue permeability (Bouchama et al., 2022). Unregulated epithelial permeability can lead to endotoxin translocation and an inflammatory response (Garcia et al., 2022). A primary response to heat stress is to increase cutaneous and subcutaneous tissue blood flow (Heinonen et al., 2011), optimizing convective heat transfer and the cooling of blood within the dermal and epidermal circulation by sweat evaporation (González‐Alonso, 2012; Kenney & Havenith, 1993). In parallel to the increase in cutaneous and muscle blood flow (Heinonen et al., 2011; Koch Esteves et al., 2024), blood flow in the splanchnic organs decreases during systemic hyperthermia, causing gut hypoperfusion and ischaemia (Rowell, 1974; Rowell et al., 1968). Further to this cardiovascular‐driven response, it remains unclear whether hyperthermia directly impacts upon the gut epithelium itself (Hall et al., 2001) or whether the reduced blood flow and therefore oxygen delivery to the gut facilitates damaging intestinal nitrosative and oxidative stress (Lambert et al., 2002; Oliver et al., 2012). Regardless of origin, these mechanisms increase the permeability of the gut in heat‐stressed humans via a degraded integrity of tight junctions (Dokladny et al., 2006, 2016), and it is this pathway that inadvertently enables an endotoxin leak from the gut lumen into the portal and then systemic circulation (Garcia et al., 2022). When liver clearance cannot mitigate the systemic endotoxin increase (Garcia et al., 2022), an inflammatory response occurs, impairing immune function and multiple organ systems, with these outcomes being associated with heat illness (Leon & Bouchama, 2015; Leon & Helwig, 2010).

An abundance of experimental research has reported robustly that hyperthermia following exercise–heat stress induces intestinal epithelial injury, as quantified via changes in intestinal fatty acid binding protein (iFABP) (Foster et al., 2023; Lee et al., 2024; Walter, Gibson, et al., 2021) and/or increases in intestinal permeability directly (Costa et al., 2017, 2019; Snipe et al., 2018). Increased iFABP has been demonstrated to be strongly indicative of intestinal barrier injury (Schellekens et al., 2014), with the presence of circulating iFABP being reflective of epithelial dysfunction and the potential for increases in gut permeability. The short half‐life of iFABP [∼11 min (Thuijls et al., 2011)] makes it a particularly relevant marker for determining rapid and sequential changes in permeability in response to incremental stress, such as hyperthermia. The use of this biomarker is also pertinent given the time course of the circulating iFABP, and the responses of cytokines and chemokines to graded passive hyperthermia have not been characterized comprehensively in the literature. Thus, temperature‐related thresholds eliciting an iFABP and/or inflammatory response during passive hyperthermia are unknown, in part because many experiments evaluate outcomes based upon before–after comparisons of changes, without control trials to quantify the normal biological and methodological variability (Roca Rubio et al., 2021; Walter, Watt, et al., 2021). Passive hyperthermia can cause intestinal injury; however, the response has been described incompletely and is likely to be more complex than a linear temperature‐dependent relationship (Laitano et al., 2019). For example, passive hyperthermia can modestly increase gut permeability (Walter, Watt, et al., 2021), although the magnitude of response is less than that of exercise‐related hyperthermia even when core temperatures are identical (Walter, Watt, et al., 2021). Although this suggests an influence of hyperthermia, the greater increase in permeability during exercise highlights that other factors beyond increased temperature also influence the resilience of the gut to injury.

It is known that the magnitude of passive hyperthermia experienced by the human body is proportional to the magnitude of thermal impulse imposed upon it (Chiesa et al., 2016; Heinonen et al., 2011; Koch Esteves et al., 2024; Watanabe et al., 2024). In the context of the present experiment, in which different body surface areas are heated to elicit time‐matched magnitudes of hyperthermia, the understanding that the greatest increase in regional and core body temperatures occurs when the whole body is passively heated relative to local passive heating is most pertinent (Watanabe et al., 2024). Presently, the subsequent influence of differing magnitudes of prolonged passive hyperthermia on intestinal epithelial injury and circulating cytokines and chemokines responses at a group and individual level is unknown. This is noteworthy and is a critical construct to address, given the potential implications of understanding hyperthermic thresholds for intestinal injury for heat illness and to guide safe and effective heat therapy interventions (Brunt & Minson, 2021; Garcia et al., 2022).

This experiment therefore aimed to quantify the impact of 2.5 h of differing magnitudes of passive hyperthermia on intestinal epithelial injury and to evaluate subsequent changes in cytokine and chemokine responses. Different magnitudes of hyperthermia were elicited by heating different body surface areas; specifically, single‐leg, two‐leg and whole‐body hyperthermia were compared with normothermic control conditions. It was hypothesized that of all the heating protocols, passive whole‐body hyperthermia would elicit the highest core temperatures and cause the greatest intestinal epithelial injury. Additionally, the greatest increase in circulating cytokine, chemokine and growth factor concentrations would be associated with the greatest magnitude of hyperthermia.

## 2 MATERIALS AND METHODS

### 2.1 Participants

Eight healthy males (age 29 ± 11 years, height 179 ± 7 cm and body mass 73 ± 10 kg) participated in the study. The sample size was estimated as appropriate a priori based upon α = 0.05, power = 0.8 in a repeated‐measures design containing four conditions and four time points where, in keeping with previous exercise–heat stress research (Snipe et al., 2018), a moderate effect size was anticipated for iFABP. The study was approved by the Brunel University of London Research Ethics Committee (6237‐A‐Jun/2017‐7569‐2) and was carried out in accordance with the *Declaration of Helsinki*. Written informed consent was obtained from all participants prior to commencement of the study. No female participants volunteered to participate in the experiment despite recruitment being open to both sexes. The participants were as a minimum recreationally active, but not training for high‐performance sport, they were free of chronic health disorders/diseases, including gastrointestinal disorders such as irritable bowel syndrome/colitis, and they were not taking prescription medications or NSAID medications (48 h prior to any experimental visit). Participants had no previous history or heat illness, had not engaged in heat training in the preceding 3 months, were not regular sauna or hot tub users and were therefore not considered to be heat acclimatized.

### 2.2 Experimental design

The present study was part of a larger investigation evaluating human circulatory control during hyperthermia, the procedures of which are described in detail elsewhere (Watanabe et al., 2024). Participants visited the laboratory on four occasions separated by >3 days, undergoing four counterbalanced protocols: (1) no heating (CON); (2) one‐leg heating (OLH); (3) two‐leg heating (TLH); and (4) whole‐body heating (WBH). In contrast to the original experimental protocol (Watanabe et al., 2024), all data for CON, OLH and TLH trials that are presented in this manuscript are abridged at 2.5 h to time match the WBH trial, given that participants reached their limit of heat tolerance after 2.5 h (2.0 h for one participant).

For each experimental visit, participants arrived at the laboratory postprandial at 08.00 h having abstained from strenuous exercise and alcohol intake for 24 h and caffeine consumption for 12 h before the commencement of the protocol. Nude body mass was measured post‐void in private, and participants then entered an environmental chamber set at 23°C (relative humidity 45%–55%) to rest in a supine position on a custom bed for the duration of the visit. Following instrumentation and the recording of baseline measurements, participants were fitted with a water‐perfused garment wrapped in a survival blanket to cover the skin surface over the body segment(s) corresponding to the trial being undertaken. The garment was connected to a thermostatically controlled water circulator (F‐34; Julabo, Germany), which continuously circulated hot water (outlet temperature = 50°C). Body temperatures were recorded continuously. Blood samples were also obtained every 30 min. To avoid a potential confounding effect of dehydration prior to commencing the experimental protocol, participants were instructed to consume 10 ml (kg body mass)^−1^ of water in the evening prior to attending the laboratory and to consume this volume of water again on waking on the morning of attendance to facilitate euhydration. Based upon pilot data, participants subsequently ingested prescribed volumes of room‐temperature water during the heating protocols to maintain euhydration (i.e., 0.1 ± 0.2, 0.3 ± 0.1 and 0.9 ± 0.1 L h^−1^ during OLH, TLH and WBH, respectively). Full details associated with the lack of changes in body mass and blood volumes have been presented previously, with the change in body mass being negligible during all trials (Watanabe et al., 2024: table 1).

### 2.3 Haematological variables

Venous blood samples were taken from a superficial antecubital vein via a venous cannula. Haemoglobin concentration was assessed via the azide methaemoglobin method (HemoCue Hb 201+ System; HemoCue AB, Sweden), and haematocrit was measured in quadruplicate using standard sodium‐heparinized capillary tubes (micro‐haematocrit tubes; Hawksley, UK), centrifugation (5 min; HaematoSpin 1400; Hawksley, UK) and microscopy‐assisted quantification. The percentage changes in blood, red cell and plasma volumes were calculated from the haemoglobin and haematocrit values (Dill & Costill, 1974). Absolute changes in blood, red cell and plasma volumes (in litres) were then estimated using established equations (Sawka et al., 1992). These data were used to correct for individual changes in plasma volume at each sample time point. As reported by Watanabe et al. (2024), neglible changes in these markers were observed, except for a difference in plasma volume between OLH and WBH only, at 2.5 h (Watanabe et al., 2024). Circulating anti‐inflammatory cytokines (IL‐4, IL‐10, IL‐13 and IL‐1RA) and pro‐inflammatory cytokines (IL‐1α, IL‐1β, IL‐6, IL‐12p40, IL‐12p70, IL‐17A, IFNγ, TNFα and TNFβ), anti‐inflammatory chemokines [MDC (CCL22)], pro‐inflammatory chemokines [eotaxin, GRO (CXCL1), IP‐10 (CXCL10), MCP‐1 (CCL2), MCP‐3 (CCL7), MIP‐1α (CCL3) and MIP‐1β (CCL4)] and growth factors (EGF, FGF‐2, Flt‐3L, fractalkine, G‐CSF, IFNα2, IL‐2, IL‐3, IL‐5, IL‐7, IL‐9, IL‐15, TGF‐α, VEGF and sCD40L) were analysed in serum using a Luminex multibead panel (MILLIPLEX MAP Premixed 38 Plex Human Cytokine/Chemokine Magnetic Bead Panel; Merck, USA). Plasma iFABP concentration was analysed using an enzyme‐linked immunosorbent assay (ELISA; Hycult Biotech, USA). Analyses were conducted in accordance with the manufacturer's directions, with concentrations corrected for changes in plasma volume (Gibson et al., 2014). The coefficient of variation for iFABP was 2.8%. All coefficients of variation for chemokines, cytokines and growth factors analysed were ≤3.0%, except for MDC (6.5%).

### 2.4 Temperature variables

Core temperature (*T*_core_) was assessed via a rectal probe (RET‐1; Physitemp Instruments, USA) inserted 15 cm past the anal sphincter. Skin temperature was recorded from four sites (chest, arm, thigh and calf) from surface thermistors affixed using thermoneutral medical tape (IT‐18; Physitemp Instruments, USA), with mean skin temperature (*T*_sk_) subsequently calculated using a standard weighted formula (Ramanathan, 1964). Mean body temperature (*T*_body_) was calculated from *T*_core_ and *T*_skin_ (Hardy et al., 1938). Temperature of the vastus lateralis muscle (*T*_m_) of the right/heated thigh was measured using a thermistor (T‐204f; Physitemp Instruments, USA) inserted through an 18‐gauge cannula ∼3 cm below the skin surface into the mid‐portion of the muscle. The *T*_core_ probe and *T*_m_ and *T*_sk_ thermistors were connected to a thermocouple meter (TC‐2000; Sable Systems, USA). All data were sampled at 1000 Hz using a data acquisition unit (Powerlab 16/30; ADInstruments, Australia).

### 2.5 Statistical analysis

Differences in measured variables were assessed using a two‐way repeated‐measures ANOVA, with the main effects being trial (CON, OLH, TLH and WBH) and time (0, 0.5, 1.0, 1.5, 2.0 and 2.5 h). For iFABP, the main effect of time was assessed on four occasions (0, 1.0, 2.0 and 2.5 h), and for cytokines/chemokines, the main effect of time was assessed on three occasions [0, 1.5 and 2.5 h (WBH)/protocol end (CON, OLH and TLH)]. Bonferroni's method was used as a *post hoc* test. Total area under the curve (Narang et al., 2020) and change (∆), calculated as the difference between the first and final time point, were analysed using one‐way ANOVA (CON, OLH, TLH and WBH), with Bonferroni's method used as a *post hoc* test. Subgroup analysis [i.e., responses in those who did report a change in iFABP after 2.5 h (∆ greater than baseline + 2 SD) versus those who did not after 2.5 h] was also conducted via a two‐way mixed‐design ANOVA, with Bonferroni's method used *post hoc*. Statistical analyses were performed using IBM SPSS Statistics (version 28; IBM, USA). Values of *P* < 0.05 were considered significant. Data are reported as means ± SD unless otherwise stated.

## 3 RESULTS

### 3.1 Temperature responses

**FIGURE 1:**
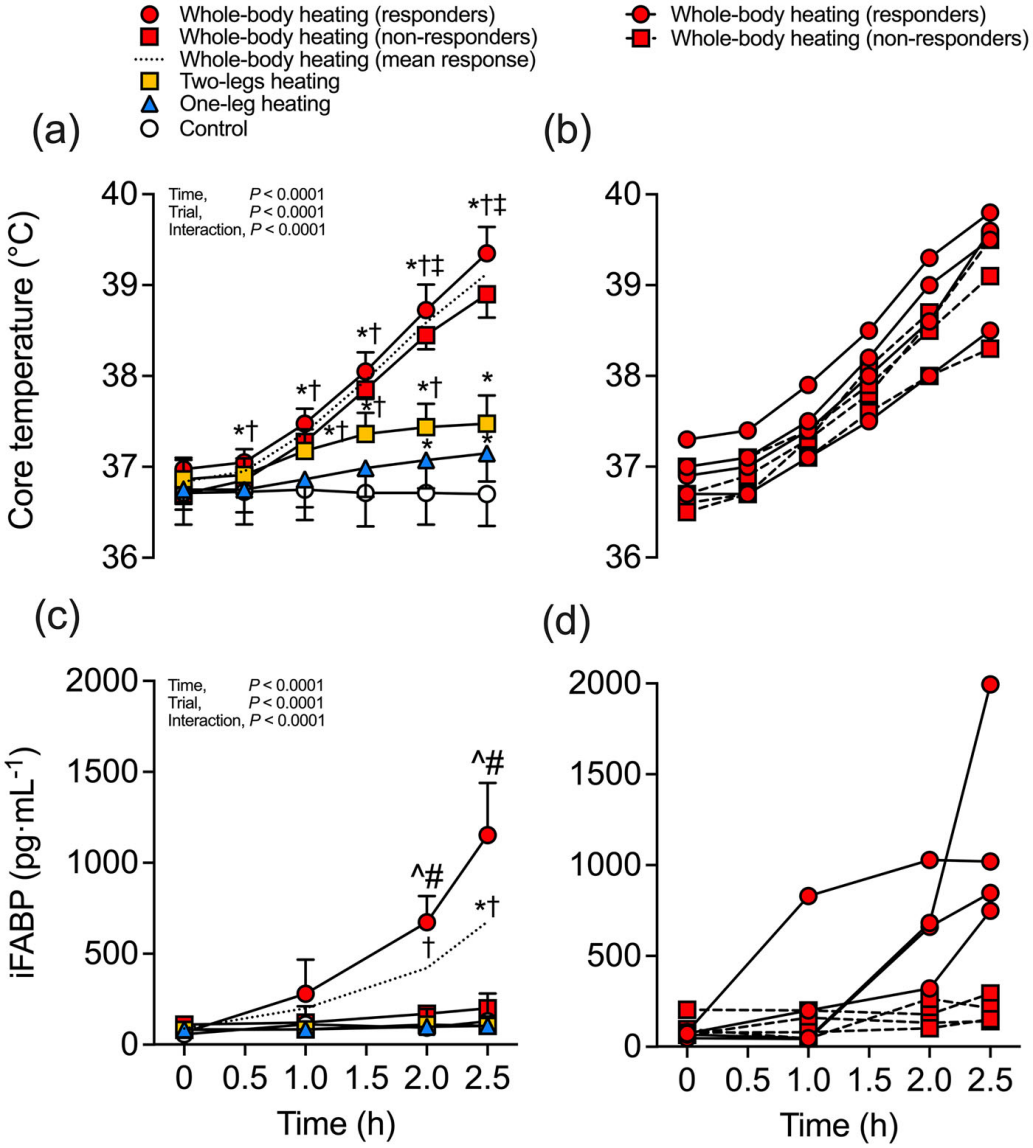
Core temperature and iFABP responses to the passive hyperthermia protocols. (a) Core temperature for all trials, including responses of iFABP responders (red circles) and non‐responders (red squares), in addition to the mean response during WBH (dotted line). (b) Individual core temperatures during WBH. (c) iFABP concentration for all trials, including responses of iFABP responders (red circles) and non‐responders (red squares) during WBH, in addition to the mean response (dotted line). (d) Individual iFABP concentrations during WBH. Data are means ± SD for eight participants. *P < 0.05 versus control; †*P* < 0.05 versus single‐leg heating; ‡*P* < 0.05 versus two‐leg heating, ^*P* < 0.05 versus 0 and 1 h within trial, #*P* < 0.05 versus WBH (non‐responders) at the corresponding time point. Abbreviations: iFABP, intestinal fatty acid binding protein; WBH, whole‐body heating.

Main and interaction effects for temperature responses were induced successfully across trials (*P* < 0.05), full details of which have been published previously (Watanabe et al., 2024). In summary, from baseline, *T*_core_ (grand mean 36.8°C ± 0.3°C) and *T*_m_ (baseline grand mean 34.4°C ± 1.1°C) increased in OLH (+0.4°C ± 0.2°C and +3.4°C ± 1.2°C), TLH (+0.6°C ± 0.2°C and +3.4°C ± 1.3°C) and WBH (+2.3°C ± 0.4°C and +6.0°C ± 1.7°C), respectively (*P* < 0.05), but were unchanged in CON (0.0°C ± 0.2°C and −1.2°C ± 0.7°C). Within WBH, a *T*_core_ of 37.4°C ± 0.3°C (+0.6°C) was observed at 1 h, with increases to 38.6°C ± 0.4°C (+1.7°C) at 2 h and to 39.2°C ± 0.6°C (+2.3°C) at 2.5 h (Figure 1). From baseline, *T*_skin_ (grand mean 31.3°C ± 0.6°C) and *T*_body_ (baseline grand mean 35.7°C ± 0.2°C) increased in OLH (+4.3°C ± 0.7°C and +1.2°C ± 0.2°C), TLH (+4.2°C ± 0.9°C and +1.3°C ± 0.2°C) and WBH (+9.0°C ± 1.1°C and +3.6°C ± 0.4°C), respectively (*P* < 0.05), but were unchanged in CON (−0.5°C ± 0.4°C and −0.1°C ± 0.1°C).

### 3.2 iFABP and cytokine and chemokine responses

When examining absolute concentrations, despite significant main effects for condition, time and their interaction, there were no *post hoc* differences observed in iFABP concentration (*P* < 0.05). The ∆iFABP and total area under the curve for iFABP also reported main effects (*P* < 0.05), but no *post hoc* differences. There were no significant differences at a main or interaction effect level for any cytokines (Table 1), chemokines (Table 2) or growth factors (Table 3).

**TABLE 1:** Circulating cytokine concentrations (in picograms per millilitre) during 2.5 h of whole‐body, two‐leg or one‐leg heating or no heating (control).

		Time (h)				
Cytokine	Conditions	0	1.5	2.5	Factor	*P*‐value
Anti‐inflammatory cytokines						
IL‐4						
	WBH	28 ± 10	30 ± 11	28 ± 12	Time	*P* = 0.712
	TLH	25 ± 14	25 ± 8	21 ± 13	Trial	*P* = 0.061
	OLH	25 ± 12	19 ± 12	22 ± 16	Interaction	*P* = 0.529
	Control	22 ± 12	22 ± ± 12	21 ± 17		
IL‐10						
	WBH	17.5 ± 21.6	19.5 ± 26.0	19.6 ± 28.1	Time	*P* = 0.336
	TLH	16.7 ± 21.6	18.8 ± 25.2	17.5 ± 24.4	Trial	*P* = 0.387
	OLH	8.4 ± 6.7	7.5 ± 5.1	9.3 ± 6.0	Interaction	*P* = 0.395
	Control	16.3 ± 22.5	16.6 ± 22.9	17.8 ± 24.3		
IL‐13						
	WBH	39 ± 53	40 ± 54	37 ± 52	Time	*P* = 0.941
	TLH	32 ± 46	26 ± 32	41 ± 51	Trial	*P* = 0.834
	OLH	36 ± 53	31 ± 57	29 ± 54	Interaction	*P* = 0.961
	Control	33 ± 53	39 ± 54	38 ± 54		
IL‐1RA						
	WBH	52 ± 104	59 ± 118	51 ± 105	Time	*P* = 0.979
	TLH	44 ± 93	31 ± 52	47 ± 99	Trial	*P* = 0.841
	OLH	51 ± 103	49 ± 108	55 ± 116	Interaction	*P* = 0.997
	Control	51 ± 109	56 ± 120	51 ± 113		
Pro‐inflammatory cytokines						
IL‐1α						
	WBH	6.7 ± 6.5	5.7 ± 5.1	5.1 ± 4.3	Time	*P* = 0.385
	TLH	4.4 ± 4.1	5.7 ± 5.4	5.8 ± 5.3	Trial	*P* = 0.545
	OLH	6.4 ± 6.6	4.2 ± 3.3	4.4 ± 3.0	Interaction	*P* = 0.136
	Control	4.7 ± 3.9	5.2 ± 4.3	4.4 ± 4.9		
IL‐1β						
	WBH	2.1 ± 0.2	2.1 ± 0.1	2.2 ± 0.1	Time	*P* = 0.640
	TLH	2.0 ± 0.0	2.1 ± 0.1	2.0 ± 0.1	Trial	*P* = 0.124
	OLH	2.1 ± 0.2	2.0 ± 0.0	2.0 ± 0.1	Interaction	*P* = 0.235
	Control	2.0 ± 0.0	2.0 ± 0.1	2.0 ± 0.2		
IL‐6						
	WBH	17 ± 23	18 ± 22	18 ± 23	Time	*P* = 0.1233
	TLH	41 ± 50	44 ± 59	51 ± 62	Trial	*P* = 0.4884
	OLH	25 ± 18	23 ± 16	27 ± 17	Interaction	*P* = 0.3891
	Control	39 ± 47	44 ± 56	45 ± 59		
IL‐12p40						
	WBH	43 ± 54	51 ± 69	48 ± 71	Time	*P* = 0.336
	TLH	16 ± 27	29 ± 63	27 ± 57	Trial	*P* = 0.468
	OLH	32 ± 69	26 ± 57	26 ± 54	Interaction	*P* = 0.175
	Control	22 ± 41	25 ± 52	13 ± 21		
IL‐12p70						
	WBH	33 ± 70	30 ± 66	22 ± 41	Time	*P* = 0.473
	TLH	16 ± 27	29 ± 63	27 ± 57	Trial	*P* = 0.446
	OLH	32 ± 69	26 ± 57	26 ± 54	Interaction	*P* = 0.445
	Control	22 ± 41	25 ± 52	13 ± 21		
IL‐17A						
	WBH	10.6 ± 17.3	10.8 ± 17.2	8.2 ± 10.1	Time	*P* = 0.391
	TLH	6.9 ± 7.7	9.4 ± 15.7	9.6 ± 13.9	Trial	*P* = 0.423
	OLH	10.8 ± 16.8	7.7 ± 13.6	8.2 ± 14.8	Interaction	*P* = 0.266
	Control	7.4 ± 10.7	8.4 ± 11.1	4.6 ± 4.1		
IFNγ						
	WBH	19 ± 32	23 ± 41	15 ± 22	Time	*P* = 0.514
	TLH	12 ± 13	18 ± 32	20 ± 30	Trial	*P* = 0.541
	OLH	22 ± 39	18 ± 33	18 ± 30	Interaction	*P* = 0.291
	Control	15 ± 20	17 ± 25	10 ± 9		
TNFα						
	WBH	6.7 ± 3.8	6.0 ± 1.7	5.3 ± 1.8	Time	*P* = 0.701
	TLH	5.1 ± 0.7	5.3 ± 2.5	5.4 ± 2.2	Trial	*P* = 0.257
	OLH	6.0 ± 2.7	5.1 ± 1.6	5.7 ± 1.6	Interaction	*P* = 0.657
	Control	4.7 ± 1.5	4.8 ± 1.9	5.0 ± 2.1		
TNFβ						
	WBH	11 ± 22	11 ± 23	11 ± 22	Time	*P* = 0.972
	TLH	10 ± 21	7 ± 12	10 ± 21	Trial	*P* = 0.910
	OLH	11 ± 22	11 ± 23	11 ± 24	Interaction	*P* = 0.999
	Control	11 ± 23	11 ± 24	11 ± 23		

*Note*: Data are means ± SD for seven participants.

Abbreviations: OLH, one‐leg heating; TLH, two‐leg heating; WBH, whole‐body heating.

**TABLE 2:** Circulating chemokine concentrations (in picograms per millilitre) during 2.5 h of whole‐body, two‐leg or one‐leg heating or no heating (control).

		Time (h)				
Chemokine	Conditions	0	1.5	2.5	Factor	*P*‐value
Anti‐inflammatory chemokines						
MDC (CCL22)						
	WBH	898 ± 226	1096 ± 404	928 ± 345	Time	*P* = 0.427
	TLH	846 ± 210	772 ± 210	804 ± 247	Trial	*P* = 0.365
	OLH	956 ± 279	840 ± 290	832 ± 259	Interaction	*P* = 0.196
	Control	901 ± 260	803 ± 310	733 ± 294		
Pro‐inflammatory chemokines						
Eotaxin						
	WBH	91 ± 76	75 ± 47	63 ± 47	Time	*P* = 0.148
	TLH	76 ± 53	68 ± 60	57 ± 31	Trial	*P* = 0.356
	OLH	84 ± 76	58 ± 42	69 ± 47	Interaction	*P* = 0.721
	Control	75 ± 67	62 ± 39	49 ± 27		
GRO (CXCL1)						
	WBH	403 ± 266	609 ± 582	364 ± 202	Time	*P* = 0.313
	TLH	333 ± 213	337 ± 236	229 ± 160	Trial	*P* = 0.654
	OLH	301 ± 215	247 ± 184	273 ± 149	Interaction	*P* = 0.383
	Control	287 ± 251	223 ± 199	205 ± 153		
IP‐10 (CXCL10)						
	WBH	227 ± 47	260 ± 91	279 ± 86	Time	*P* = 0.724
	TLH	370 ± 388	358 ± 396	227 ± 40	Trial	*P* = 0.841
	OLH	284 ± 159	248 ± 182	261 ± 208	Interaction	*P* = 0.780
	Control	282 ± 66	269 ± 114	334 ± 208		
MCP‐1 (CCL2)						
	WBH	238 ± 72	233 ± 95	227 ± 77	Time	*P* = 0.335
	TLH	210 ± 60	184 ± 81	166 ± 48	Trial	*P* = 0.122
	OLH	222 ± 81	158 ± 91	189 ± 110	Interaction	*P* = 0.699
	Control	191 ± 94	174 ± 63	210 ± 111		
MCP‐3 (CCL7)						
	WBH	68 ± 106	74 ± 114	70 ± 101	Time	*P* = 0.986
	TLH	62 ± 99	51 ± 63	63 ± 101	Trial	*P* = 0.962
	OLH	63 ± 107	60 ± 104	61 ± 104	Interaction	*P* = 0.999
	Control	64 ± 104	72 ± 116	71 ± 112		
MIP‐1α (CCL3)						
	WBH	28 ± 24	30 ± 26	24 ± 25	Time	*P* = 0.815
	TLH	23 ± 19	25 ± 23	30 ± 21	Trial	*P* = 0.973
	OLH	27 ± 24	25 ± 22	26 ± 22	Interaction	*P* = 0.647
	Control	24 ± 20	28 ± 23	24 ± 20		
MIP‐1β (CCL4)						
	WBH	26 ± 32	25 ± 27	20 ± 22	Time	*P* = 0.824
	TLH	19 ± 17	19 ± 27	22 ± 25	Trial	*P* = 0.443
	OLH	25 ± 29	22 ± 24	24 ± 25	Interaction	*P* = 0.858
	Control	21 ± 24	22 ± 24	19 ± 17		

*Note*: Data are means ± SD for seven participants.

Abbreviations: OLH, one‐leg heating; TLH, two‐leg heating; WBH, whole‐body heating.

**TABLE 3:** Circulating growth factors (in picograms per millilitre) during 2.5 h of whole‐body, two‐leg or one leg heating or no heating (control).

		Time (h)				
Growth factor	Conditions	0	1.5	2.5	Factor	*P*‐value
EGF						
	WBH	51 ± 55	40 ± 36	31 ± 35	Time	*P* = 0.228
	TLH	41 ± 36	30 ± 28	28 ± 33	Trial	*P* = 0.848
	OLH	43 ± 40	31 ± 35	28 ± 33	Interaction	*P* = 0.988
	Control	42 ± 35	37 ± 34	27 ± 35		
FGF‐2						
	WBH	77 ± 79	70 ± 62	63 ± 49	Time	*P* = 0.399
	TLH	57 ± 39	62 ± 64	66 ± 74	Trial	*P* = 0.374
	OLH	68 ± 87	60 ± 63	64 ± 64	Interaction	*P* = 0.574
	Control	61 ± 59	68 ± 67	51 ± 29		
Flt‐3L						
	WBH	38 ± 40	38 ± 36	35 ± 26	Time	*P* = 0.652
	TLH	29 ± 16	31 ± 32	33 ± 31	Trial	*P* = 0.082
	OLH	35 ± 36	29 ± 27	32 ± 26	Interaction	*P* = 0.630
	Control	30 ± 22	31 ± 28	24 ± 13		
Fractalkine						
	WBH	244 ± 444	197 ± 327	158 ± 246	Time	*P* = 0.527
	TLH	119 ± 150	199 ± 345	199 ± 345	Trial	*P* = 0.732
	OLH	220 ± 410	164 ± 277	177 ± 296	Interaction	*P* = 0.388
	Control	137 ± 186	188 ± 257	125 ± 95		
G‐CSF						
	WBH	66 ± 76	70 ± 85	64 ± 83	Time	*P* = 0.770
	TLH	55 ± 63	61 ± 73	64 ± 80	Trial	*P* = 0.940
	OLH	62 ± 90	56 ± 79	68 ± 84	Interaction	*P* = 0.578
	Control	60 ± 77	68 ± 87	57 ± 61		
IFNa2						
	WBH	9.6 ± 3.3	8.6 ± 3.2	7.8 ± 4.3	Time	*P* = 0.548
	TLH	8.0 ± 5.8	8.3 ± 4.9	6.4 ± 3.3	Trial	*P* = 0.828
	OLH	9.5 ± 7.8	5.9 ± 3.3	9.0 ± 4.8	Interaction	*P* = 0.427
	Control	7.2 ± 4.2	7.6 ± 3.0	8.0 ± 6.1		
IL‐2						
	WBH	2.5 ± 2.4	2.0 ± 0.7	1.8 ± 0.2	Time	*P* = 0.376
	TLH	1.6 ± 0.0	2.1 ± 1.2	2.2 ± 1.6	Trial	*P* = 0.632
	OLH	2.3 ± 1.8	1.7 ± 1.2	1.7 ± 1.3	Interaction	*P* = 0.452
	Control	1.9 ± 1.0	2.0 ± 1.2	1.6 ± 0.1		
IL‐3						
	WBH	1.3 ± 1.1	1.3 ± 1.1	1.3 ± 1.0	Time	*P* = 0.587
	TLH	1.2 ± 0.8	1.2 ± 0.9	1.2 ± 0.9	Trial	*P* = 0.364
	OLH	1.2 ± 0.8	0.7 ± 0.3	0.8 ± 0.3	Interaction	*P* = 0.332
	Control	1.1 ± 0.6	1.2 ± 0.8	1.3 ± 1.0		
IL‐5						
	WBH	4.2 ± 4.9	4.2 ± 4.3	4.1 ± 4.1	Time	*P* = 0.837
	TLH	4.5 ± 4.7	3.5 ± 2.0	4.0 ± 4.2	Trial	*P* = 0.987
	OLH	4.0 ± 4.5	3.6 ± 4.5	4.1 ± 4.9	Interaction	*P* = 0.996
	Control	4.0 ± 4.4	4.1 ± 4.7	4.0 ± 4.5		
IL‐7						
	WBH	6.0 ± 6.0	5.8 ± 4.6	6.6 ± 7.3	Time	*P* = 0.554
	TLH	5.7 ± 7.3	3.5 ± 2.6	5.9 ± 8.7	Trial	*P* = 0.337
	OLH	5.4 ± 4.5	4.4 ± 6.8	6.0 ± 9.1	Interaction	*P* = 0.950
	Control	6.7 ± 7.6	8.3 ± 10.2	7.3 ± 6.1		
IL‐9						
	WBH	6.5 ± 9.7	5.4 ± 6.4	4.3 ± 4.1	Time	*P* = 0.313
	TLH	3.8 ± 3.4	5.4 ± 7.1	5.4 ± 6.4	Trial	*P* = 0.506
	OLH	5.4 ± 7.2	3.3 ± 4.8	3.8 ± 5.8	Interaction	*P* = 0.441
	Control	4.9 ± 5.5	4.7 ± 4.9	3.3 ± 3.0		
IL‐15						
	WBH	9.1 ± 9.7	10.4 ± 12.0	9.3 ± 10.3	Time	*P* = 0.576
	TLH	8.2 ± 8.5	8.8 ± 10.1	10.0 ± 10.0	Trial	*P* = 0.359
	OLH	8.8 ± 9.3	4.3 ± 2.9	4.6 ± 3.1	Interaction	*P* = 0.287
	Control	8.3 ± 9.6	8.1 ± 8.8	8.4 ± 9.7		
TGF‐α						
	WBH	2.4 ± 1.9	2.1 ± 0.8	2.1 ± 0.6	Time	*P* = 0.240
	TLH	1.7 ± 0.2	2.1 ± 1.1	2.5 ± 2.2	Trial	*P* = 0.230
	OLH	2.2 ± 1.4	1.8 ± 0.5	2.0 ± 1.0	Interaction	*P* = 0.479
	Control	1.8 ± 0.3	2.1 ± 1.2	1.7 ± 0.1		
VEGF						
	WBH	125 ± 167	118 ± 145	99 ± 124	Time	*P* = 0.561
	TLH	94 ± 85	101 ± 149	123 ± 142	Trial	*P* = 0.856
	OLH	119 ± 173	97 ± 134	105 ± 138	Interaction	*P* = 0.243
	Control	110 ± 130	115 ± 129	80 ± 68		
sCD40L						
	WBH	1183 ± 1440	734 ± 832	356 ± 482	Time	*P* = 0.115
	TLH	1041 ± 1288	619 ± 930	330 ± 550	Trial	*P* = 0.967
	OLH	1047 ± 1542	565 ± 1158	531 ± 639	Interaction	*P* = 0.974
	Control	1126 ± 1391	565 ± 1064	318 ± 396		

*Note*: Data are means ± SD for seven participants.

Abbreviations: OLH, one‐leg heating; TLH, two‐leg heating; WBH, whole‐body heating.

### 3.3 Secondary analyses of individual responses

**FIGURE 2:**
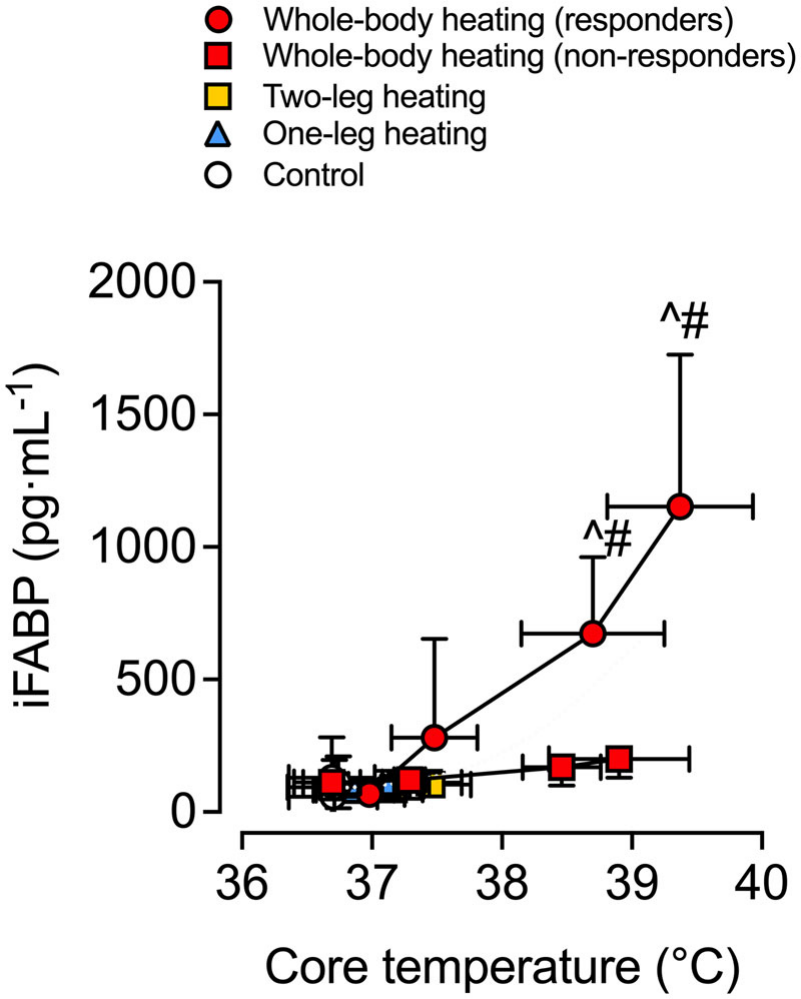
Relationship between the change in core temperature and change in iFABP across protocols. Data are presented as the mean (symbol), with error bars depicting standard deviations for both dependent variables. ^*P* < 0.05, change from 0 h within the WBH (responders) group; #*P* < 0.05, difference between WBH (responders) and WBH (non‐responders) subgroups. Abbreviations: iFABP, intestinal fatty acid binding protein; WBH, whole‐body heating.

Examination of the individual responses during WBH highlighted that the iFABP concentration had increased from baseline (*F* = 8.2, *P* = 0.001) and was greater at 2 and 2.5 h (*F* = 5.8, *P* = 0.006) in the subset of responders (*n* = 4; 2 h = 673 ± 289 pg ml^−1^; 2.5 h = 1153 ± 573 pg ml^−1^) in comparison to non‐responders who reported no change from baseline (*n* = 4; 2 h = 170 ± 70 pg ml^−1^; 2.5 h = 200 ± 70 pg ml^−1^; Figure 1c,d). Core temperature responses (Figure 1a,b) were not different between the responder and non‐responder subgroups (peak *T*_core_: responders = 39.4°C ± 0.6°C, +2.4°C ± 0.4°C; non‐responders = 38.9°C ± 0.5°C, +2.2°C ± 0.5°C; *P* > 0.05). The relationship between the change in core temperature and iFABP for each group/subgroup is illustrated in Figure 2. No cytokine, chemokine or growth factor demonstrated any inter‐individual response. Given the lack of statistical difference, regression analyses were not performed on these variables.

## 4 DISCUSSION

The primary finding arising from this experiment is that prolonged passive whole‐body but not lower‐limb hyperthermia can cause intestinal epithelial injury in some individuals without systemic inflammation in any participant. As hypothesized and by design, the passive hyperthermia trials successfully elicited incremental core temperatures of 37.1°C ± 0.3°C in OLH, 37.5°C ± 0.3°C in TLH and 39.1°C ± 0.6°C in WBH at the end of the protocol. Notably, iFABP concentrations remained unchanged in all participants during control conditions, one‐leg or two‐legs hyperthermia, but were increased during whole‐body heating, and cytokine, chemokine and growth factor concentrations were unaltered across all passive hyperthermia trials. Of particular note is the potential identification of inter‐individual differences in response to the protocol. Fifty per cent of participants experienced increases in iFABP concentration (peak = 1153 ± 573 pg ml^−1^) during WBH, whereas the remaining cohort demonstrated no evidence of intestinal injury (peak iFABP = 200 ± 70 pg ml^−1^). This finding suggests the possibility of a ‘responder’ and ‘non‐responder’ paradigm concerning gastrointestinal injury during hyperthermia, warranting further investigation.

### 4.1 Intestinal injury and individual responses

**FIGURE 3:**
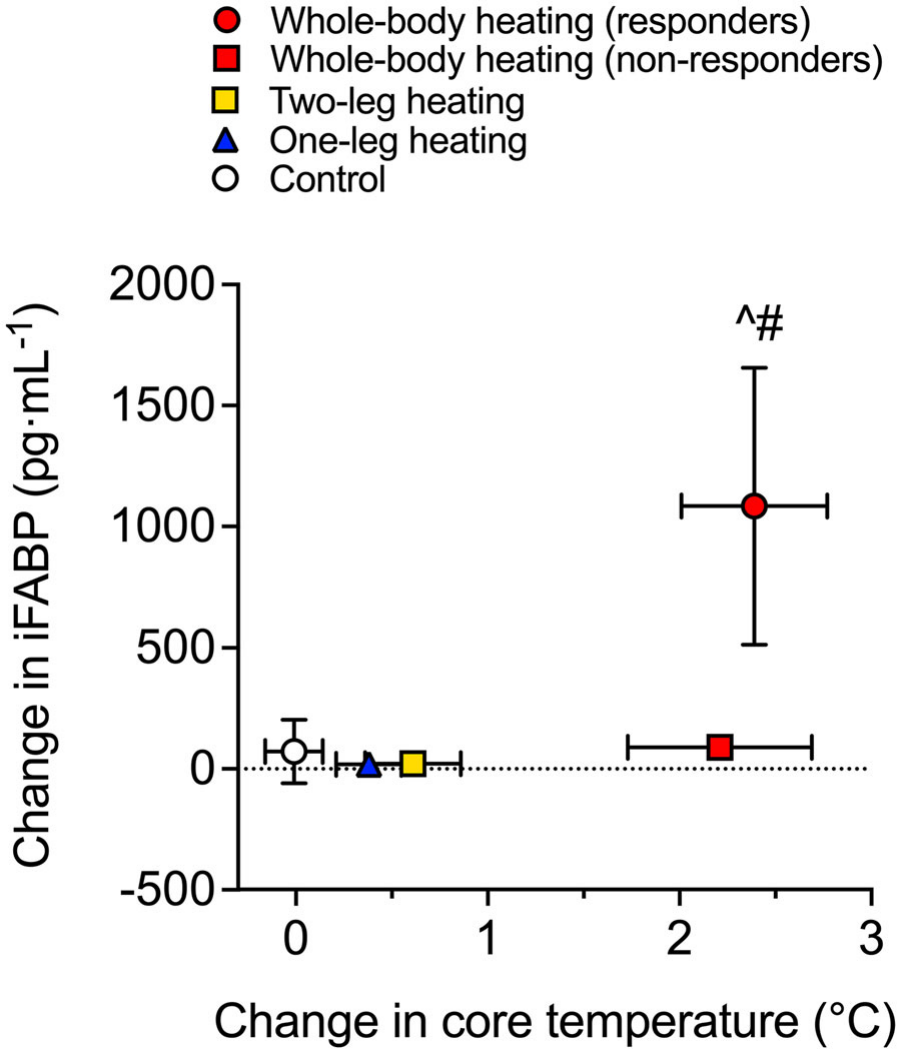
Relationship between iFABP and core temperature responses during the WBH, two‐leg and one‐leg heating and control trials. WBH is drawn to show different relationships between those responders reporting a change in iFABP concentration during the WBH trial (red circles) and those non‐responders with an unchanged iFABP concentration during the WBH trial (red squares). Data are group means ± SD for eight participants, except WBH, which is separated into responders (*n* = 4) and non‐responders (*n* = 4). ^*P* < 0.05 versus 0 and 1 h within trial, #*P* < 0.05 versus WBH (non‐responders) at the corresponding time point. Abbreviations: iFABP, intestinal fatty acid binding protein; WBH, whole‐body heating.

The impact of the passive heating protocols used in this experiment on body temperatures, central and peripheral cardiovascular and haemodynamic responses, respiratory outcomes and metabolic parameters have been reported extensively elsewhere (Watanabe et al., 2024). Further to these outcomes, it is now apparent that one‐ and two‐legs heating do not elicit intestinal injury or modify the concentration of the 38 pro‐ and anti‐inflammatory cytokines and chemokines or the growth factors measured in this experiment. In contrast, by the end of the whole‐body heating protocol substantial increases in core temperature were observed (+2.3°C ± 0.4°C), with 50% of the participants demonstrating an increase in iFABP (∆1085 ± 572 pg ml^−1^; Figure 3). The onset of the increase in iFABP was observed after 2 h, when core temperatures typically associated with whole‐body heat therapy interventions were observed (38.6°C ± 0.4°C, +1.8°C ± 0.3°C). The impact of passive hyperthermia on intestinal injury and gut permeability remains understudied, with the existence of a temperature threshold for compromised gut integrity during passive heating unknown. Exposure to passive environmental heat stress but with unaltered core temperature does not increase iFABP (Sheahen et al., 2018); however, ∼3 h of intermittent sauna bathing (in conjunction with moderate dehydration) eliciting increased core temperatures (to 38.6°C) substantially increases gut permeability (Roca Rubio et al., 2021). These studies indicate an influence of hyperthermia on intestinal injury and gut permeability; however, the independence of this effect is unknown.

A notable finding is the potential identification of a responder versus non‐responder paradigm, given the disparate intestinal injury responses between participants despite comparable peak and change core temperatures. Literature describing the inter‐individual responses to passive hyperthermia are scarce, thus understanding the precedent for inter‐individual or responder versus non‐responder outcomes in gut permeability during passive hyperthermia is challenging. A recent experiment comparing alterations in gut permeability in passive (hot water immersion) versus exertional hyperthermia found that 100% of participants reported an increase in permeability during exertional hyperthermia, with 33% demonstrating an increase during passive hyperthermia despite equivalent core temperatures (39.3°C ± 0.2°C) (Walter, Watt, et al., 2021). This outcome aligns with meta‐analyses which identified that in exertional hyperthermia 100% of participants demonstrate compromised gut integrity when core temperature is ≥39.1°C (Pires et al., 2017). Interestingly, at core temperatures of 38.6°C–39.0°C, 48% of participants report increased intestinal permeability during exercise (Pires et al., 2017). In our whole‐body hyperthermia trial, at a group mean core temperature of 39.1°C only 50% of our participants demonstrated signs of intestinal injury. Taken together, these data imply that the threshold for intestinal injury might occur at a higher core temperature in passive versus exertional hyperthermia. McKenna et al. (2024) examined the impact of hyperthermia to the limits of participant tolerance (core temperature ∆+2°C) on gastrointestinal permeability, microbial translocation and systemic inflammation in young and older adults. In this experiment, hyperthermia induced via ∼70 min of low‐intensity exercise and a 50°C water‐perfused suit resulted in modest increases in intestinal permeability as assessed by lactulose:rhamnose and lipopolysaccharide binding protein, with a mild inflammatory response, but no increase in iFABP. Examination of the individual data also indicated disparate iFABP responses between participants. Specifically, 44% of their young participants and 66% of their older participants reported a pre–post increase in iFABP approaching +500 pg ml^−1^. This work provides insight into responses using an ecologically valid model; however, without relevant controls (i.e., euhydration, normothermic exercise and passive hyperthermia) and without serial biomarker measurements, the independent role of hyperthermia on these responses cannot be established conclusively. Taken together, their data and ours further question the notion that hyperthermia is a direct contributing factor in intestinal injury and increased gut permeability, with further examination of the proportional direct and indirect effects of hyperthermia on intestinal injury, gut permeability and systemic inflammation being required (Laitano et al., 2019).

### 4.2 Inflammatory and growth factor responses

The influence of hyperthermia on circulating inflammatory markers and understanding of whether a hyperthermia–inflammation dose response exists is complex and equivocal, with outcomes seeming to be contingent on many contextual factors (Welc et al., 2012). Our absence of response in circulating anti‐ and pro‐inflammatory markers and growth factors oppose some passive hyperthermia literature, which observed that healthy adults experienced a ≤3‐fold change in frequently examined cytokines (e.g., IL‐6) following whole‐body passive heating to core temperature magnitudes comparable to those elicited in the present experiment (Faulkner et al., 2017; Laing et al., 2008; Su et al., 2024). Likewise, two‐legs heating inducing a +1°C increase in core temperature might induce an inflammatory response in individuals with spinal cord injury (Hashizaki et al., 2018). However, in agreement with our work, no inflammatory response is observed when healthy participants receive two‐legs heating, probably owing to minimal changes in core temperature (Monroe et al., 2021). Given this, our study and others (Monroe et al., 2021) create uncertainty as to whether hyperthermia is a relevant regulatory mechanism. The unaltered inflammatory profile might be reflective of the healthy cohort tested in our study, because it is known that in cases of exertional heat stroke without liver damage, endotoxin is not detected in the circulation despite evidence of gastrointestinal injury. This suggests that the liver effectively clears endotoxin before a significant inflammatory response occurs (Garcia et al., 2022). Conversely, in cases with liver damage, endotoxin accumulates in the circulation, often with catastrophic consequences.

Differences in the delivery of heat stress might also be a relevant factor modulating the inflammatory response during hyperthermia. Skeletal muscle is a tissue from which cytokines are released abundantly into the circulation, with muscle contraction being a recognized stimulus for this outcome (Pedersen & Febbraio, 2008). Owing to the extensive haemodynamic assessments occurring in this study (Watanabe et al., 2024), participants were exposed to gradual heating (∼1°C h^−1^) and unable to move during our water‐perfused garment trials. In contrast, during hot water immersion experiments participants are heated more rapidly (∼2°C h^−1^) and able to move more freely. The influence of the rate and duration of passive hyperthermia on gut permeability remains an area of future investigation; however, given that muscle contraction is a potent stimulus to elicit a ≤3‐fold increase in inflammatory markers even at low intensities (Pedersen & Febbraio, 2008), the combination of muscle contraction and hyperthermia might be the true potentiating stimuli for increased circulating concentrations, as highlighted when comparisons are made between our data and those eliciting equivalent hyperthermia during exercise at low (McKenna et al., 2024) and moderate intensity (Willmott et al., 2018).

The null cytokine, chemokine and growth factor outcomes in our study do not give countenance to the proposal that heat/thermal therapy is inducing a positive adaptive response by this mechanism (Brunt & Minson, 2021). Therefore, heat/thermal therapy interventions might be effective via a more direct action on target organs and tissues or via circulating markers not measured in this experiment that are influential in orchestrating the adaptive response. In light of this, heat therapy interventions might reconsider the independent effect of hyperthermia and focus upon treatment delivery using hyperthermia and contractile activity to optimize outcomes. Although potentially negative in the context of heat/thermal therapy, the absence of an inflammatory response is positive in the context of heat illness. Specifically, these data partly mitigate concerns that hyperthermia of the magnitudes elicited in this study might be harmful to health during heat therapy interventions. An inflammatory response following heat stress can be indicative that the gut has become ‘leaky’, an outcome that is associated with manifestation of heat illness (Bouchama et al., 2022; Laitano et al., 2019). Although interpretation of inflammatory responses can be complex in the context of heat illness (Leon & Bouchama, 2015), the absence of a response following our hyperthermia protocols indicates an absence of circulating/systemic stress emanating from intestinal injury.

### 4.3 Experimental limitations and implications for future research

Although we did not measure the change in gut permeability directly, as commonly quantified by dual‐sugar tests, iFABP is strongly associated with the magnitude of gastrointestinal barrier injury, with tissue injury being the precursor to increased permeability (Schellekens et al., 2014). Quantifying changes in lipopolysaccharide or lipopolysaccharide binding protein would also have enabled a more complete characterization of the effects of passive hyperthermia on endotoxin translocation (Ogden et al., 2020). In the absence of an inflammatory response, it is assumed that no endotoxin translocation occurred during our passive hyperthermia model or that it was cleared effectively via the liver (Garcia et al., 2022). Likewise, there is potential discrepancy between local tissue cytokine/chemokine concentrations and the unchanged circulatory concentrations. For example, we have previously reported increases in VEGFα mRNA and protein in the vastus lateralis in response to prolonged one‐leg heating (Gibson et al., 2023), but the present study yielded no change in circulating VEGFα despite directly comparable protocols. Accordingly, local versus systemic factors, in addition to the role of microvesicles (Wilhelm et al., 2017), should be considered more completely. It is also acknowledged that although the experimental design enabled examination of the response to differing magnitudes of hyperthermia over the same duration of heating for each participant, the study was not able to delineate fully the independent effects of hyperthermia on increases in gut permeability at the same core temperature for each participant. Accordingly, an isothermic model might enable further insights into inter‐individual and inter‐protocol responses (Gibson et al., 2015; Mee et al., 2016). Aligned to this, maximum endogenous concentrations of our selected markers might have peaked at different time points (within and between participants) following the end of our heating period, thus future work should consider concentration kinetics during recovery and whether this alters responder/non‐responder interpretations. Finally, given that reduced splanchnic blood flow and subsequent oxidative and nitrosative stress is proposed as a central mediator of the change in permeability (Lambert et al., 2002; Oliver et al., 2012), the absence of these measurements means that we are unable to verify their respective contributions or reconcile the direct or indirect roles of core temperature on epithelial injury and increased gut permeability. It is also acknowledged that the study was not designed or powered to identify responders and non‐responders. Future experimental work to address this question and identify predisposing factors should be designed accordingly, with assessments made in diverse population groups in normal and altered physiological states (e.g., during euhydration). Given that no female participants volunteered for our study, a limitation is that we were unable to contribute insights into the speculated impact of sex, menstrual cycle phase and contraception on these responses (Flood et al., 2022; Giersch et al., 2022).

## 5 CONCLUSION

Prolonged passive whole‐body, but not lower‐limb, hyperthermia can cause intestinal epithelial injury in some individuals without indications of systemic inflammation. The core temperature of individuals who experienced intestinal injury was not different from those who did not experience injury, pointing towards a potential responder/non‐responder paradigm and an equivocal role of hyperthermia.

## AUTHOR CONTRIBUTIONS

This study was part of a comprehensive investigation focusing on the control of blood circulation during hyperthermia (Watanabe et al., 2024). José González‐Alonso, Orlando Laitano and Oliver R. Gibson conceived and designed the study with respect to the cytokine, chemokine and iFABP responses to leg(s)‐ and whole‐body hyperthermia. José González‐Alonso, Kazuhito Watanabe and Oliver R. Gibson were involved in the data collection. Orlando Laitano analysed the cytokine and chemokine data at the University of Florida at Gainesville, FL, USA, and Oliver R. Gibson the iFABP data at Brunel University of London, UK. All authors revised the manuscript for important intellectual content. All authors approved the final version of the manuscript and agree to be accountable for all aspects of the work in ensuring that questions related to the accuracy or integrity of any part of the work are appropriately investigated and resolved. All persons designated as authors qualify for authorship, and all those who qualify for authorship are listed.

## CONFLICT OF INTEREST

The authors declare no conflicts of interest.

## Data Availability

The raw, unidentified data collected throughout this study will be made available via Brunel Figshare, an online data repository database.
